# Pilot Study on the Efficacy and Safety of Long-Term Oral Imepitoin Treatment for Control of (Thunder)Storm-Associated Noise Phobia/Noise Aversion in Dogs Using an Individualized-Dose Titration Approach

**DOI:** 10.3390/ani14040545

**Published:** 2024-02-06

**Authors:** Ana C. Muñoz Amezcua, Jennifer M. Jones, Emily H. Griffith, Margaret E. Gruen

**Affiliations:** 1 Department of Clinical Sciences, College of Veterinary Medicine, North Carolina State University, Raleigh, NC 27606, USA; 2 Department of Statistics, North Carolina State University, Raleigh, NC 27606, USA; eghohmei@ncsu.edu

**Keywords:** imepitoin, storm, anxiety, anxiolytic, antiepileptic, noise, phobia, clinical trial

## Abstract

**Simple Summary:**

Imepitoin is a medication that can be used to treat anxiety in dogs. Previous studies have shown that imepitoin when given at a dose of 30 mg/kg PO BID (milligram per kilogram of body weight by mouth twice a day), can reduce noise-related anxiety in dogs. However, this dosage may be higher than needed for some dogs. We aimed to determine how safe and effective this medication is for the treatment of storm-related fear and anxiety in dogs when started at a lower dose (10 mg/kg PO BID) and, if needed, when increased to a higher dose of 20 or 30 mg/kg PO BID. We determined the three dosages were safe and reduced clinical signs of storm related fear and anxiety in this study design. More side effects were seen in the 20 mg/kg PO BID group than in the 10 mg/kg PO BID group, which supports the use of this medication at an individually titrated dose starting at 10 mg/kg PO BID.

**Abstract:**

Imepitoin is a low-affinity partial agonist for benzodiazepine binding sites of gamma-aminobutyric acid receptors with anxiolytic effects. It has been shown to reduce anxiety during noise-related events in dogs when given at 30 mg/kg PO BID, although this dose was associated with ataxia and increased appetite in some cases. The objective of this study was to assess its safety and efficacy for storm anxiety when started at 10 mg/kg PO BID and titrated to effect up to 30 mg/kg PO BID during storm season. Significant decreases in anxiety scores were seen in weekly surveys and storm logs (SLs) at 10, 20 and 30 mg/kg PO BID. Serious adverse events (AEs) were not reported in any subject. Ataxia was the most commonly reported non-serious AE (14/33), followed by increased hunger (13/33). The frequency of AEs was higher in the 20 mg/kg PO BID group than in the 10 mg/kg group PO BID. No clinically significant changes were seen in lab work pre- and post-study. In conclusion, Imepitoin given during storm season at doses ranging from 10 to 30 mg/kg PO BID reduced clinical signs of fear and anxiety during storms for the dogs in this study. These findings support the use of an individually titrated dose.

## 1. Introduction

Canine behavioral disorders are common, with a prevalence in the pet dog population of up to 85% [1]. A large proportion of these conditions, including canine storm anxiety, are fear or anxiety based [1,2,3]. Fear and anxiety, defined as “emotional states that are induced by the perception of any actual danger (fear state) or potential danger (anxiety state)” [4], are crucial emotions that can help animals survive when faced with known or potential threats [5]. These emotions become pathological when they are disproportionate to the stimulus, are generalized, or interfere with normal function [2,6,7]. In this study, we use the term “storm anxiety” to refer to a maladaptive fearful, anxious, or phobic response to storms [8]. This diagnosis is sometimes classified under the umbrella of noise aversion or noise phobia but is theorized and anecdotally reported to also occur in response to non-auditory meteorologic stimuli such as changes in barometric pressure, static electricity, odors, and flashes of lightning [1,9,10]. Not all dogs with noise aversion have storm anxiety [1,10,11]; however the presence of one condition appears to increase the risk of the other [11]. Regardless of the specific cause of dogs’ response to storms, this maladaptive fear, anxiety, or phobia adversely affects their welfare [12] and poses as a significant caregiver burden that can damage the human–animal bond, potentially leading to the euthanasia or relinquishment of the dog [13,14,15,16,17,18].

The treatment of storm anxiety consists of environmental management, behavior modification, and psychoactive medication [9,19]. Environmental management focuses on decreasing the storm’s intensity for the dog, for example, by allowing the dog to hide in an area of the house where the storm stimuli are minimized and adding noise buffers or turning the lights on to lessen the startling nature of the storm [19,20]. Behavior modification generally involves desensitization and classical counterconditioning [9,19,20] to change the dog’s emotional response to the storm from negative to positive [21,22,23]; however, the nature of storms makes this process difficult due to their unpredictability and range of potential triggers that are not easily simulated [1,9,10]. Psychoactive medications can prevent or significantly decrease the fear and anxiety experienced by dogs during storms and facilitate the implementation of behavioral modification plans [9,13,19,20,24].

When using medication to treat fear and anxiety, the frequency of administration depends, in part, on the nature of the fear-provoking stimuli [25]. For dogs with storm anxiety, the most appropriate regimen may also be affected by the region of the world in which the dog lives [8]. Dogs who live in areas where storms are frequent year round often benefit from daily medications, while others might need medication during certain seasons when storms occur more regularly [9,19] and others only need medication episodically. Medications commonly used for chronic anxiety, such as selective serotonin reuptake inhibitors (SSRIs) and tricyclic antidepressants (TCAs) require multiple weeks of administration before they have a therapeutic effect [19,20]. Other medications are given on an “as needed” or “situational” basis; these medications are typically short acting but can take over an hour to the onset of therapeutic effect [26,27], and their effectiveness is limited if owners cannot administer them ahead of a storm [20,26]. In regions of the world where there is a specific time of year during which storms occur more frequently, the daily administration of a medication with a short onset of effect makes the pharmacological treatment of storm anxiety more feasible [8].

While two medications have been approved for the treatment of noise aversion [28,29], none have been labeled for treatment of storm anxiety; however, one of these, imepitoin (Pexion^TM^, Boehringer Ingelheim Vetmedica, Ingelheim, Germany), has been evaluated for the treatment of storm anxiety in a small, randomized placebo-controlled trial [8] and in a thunderstorm-noise-induced model of fear and anxiety in dogs [30]. Imepitoin is a low-affinity partial agonist to the gamma-aminobutyric acid (GABAa) receptor with low intrinsic activity [31,32] shown to have both anticonvulsant and anxiolytic activity [30,31,32]. This medication offers the benefit of a longer half-life, requiring a twice-daily dosing [32] for managing dogs with storm anxiety, particularly during storm season [8]. As a partial agonist, it causes no sedative effects and is not associated with tolerance and dependence during long-term administration compared to full agonists (e.g., diazepam) [31]. Imepitoin is approved in the European Union (EU) and other markets (e.g., Australia and Japan) for the treatment of epilepsy at a dosage range of 10–30 mg/kg BID [33] and in both the EU and Unites States of America (USA) for noise aversion at a dose of 30 mg/kg per os (PO) bis in die (BID) [28,33].

In randomized, placebo-controlled studies, imepitoin has been shown to reduce anxiety during storms [8] and other noise-related events [34] when given at 30 mg/kg PO BID. When administered at this dosage, imepitoin was more effective than the placebo in lowing anxiety scores, but this dosage might be higher than needed for the management of storm anxiety in some dogs. For example, a case series describing the use of imepitoin at 5–30 mg/kg PO BID for anxiety-related disorders found that an average dosage of 20 mg/kg PO BID decreased anxiety scores [35]. This case series reported a low frequency of adverse effects when starting at 10 mg/kg PO BID and titrating the dose to effect [35].

The objective of the current study was to assess the use of imepitoin for the treatment of storm anxiety and fear when administered twice daily at an individually titrated dose, starting at approximately 10 mg/kg PO BID, up to approximately 30 mg/kg PO BID over a prolonged period of 12–17 weeks during storm season. We predicted that this approach would significantly decrease storm anxiety scores on a standardized scale and have a low number of adverse events (AEs) associated with administration.

## 2. Materials and Methods

This study was a single-site, prospective, open-label, dose-escalation study carried out with approval from the North Carolina State University (NCSU) Institutional Animal Care and Use Committee (Protocol #21-190-01) and followed relevant guidelines. As all of the questionnaires completed by owners involved questions specifically about the dogs, there were no experimental protocols involving humans. IRB approval was not sought because all collected data pertained to dogs, and as such, the work was categorized as Non-Human Subject Research. All owners provided written informed consent prior to the enrollment of their pet. All methods involving humans were carried out in accordance with relevant guidelines and regulations (Declaration of Helsinki), and all methods involving animals were conducted in accordance with relevant guidelines and regulations, and reported in accordance with ARRIVE guidelines (https://arriveguidelines.org; accessed on 1 July 2023). The study was carried out over the period between mid-April and mid-October, which is considered storm season in North Carolina. All dogs were client-owned and remained with their owners throughout the study. The owners gave written informed consent prior to the start of the study.

Participants were recruited via social media, national public radio advertisements, and flyers in the local area. To be eligible, dogs had to be over one year of age, weigh more than 5 kg, have lived in their home for at least one year, be healthy or with stable systemic disease, and be pain-free or taking pain medication without changes in dose for at least two weeks before inclusion. Dogs needed to have a diagnosis of storm anxiety based on a medical/behavioral history confirming storm anxiety and a minimum qualifying score of 30 on the Lincoln Sound Sensitivity Scale (LSSS) [10,36,37], as described under outcome assessments. Owners also had to confirm that they would be with their dog during enough storms in order to complete the assessments, and that they would complete all study-related questionnaires. Dogs were excluded if they were taking behavior-modifying drugs (drugs licensed as an antidepressant, antipsychotic, anxiolytic, hypnotic, mood stabilizer or stimulant in humans, dogs or other species), if they were fearful or anxious the majority of the time (such that response to storms would be difficult to distinguish from other fearful responses) or if they had a history of known epilepsy or other disease that would expose them to unacceptable risk, preclude study completion or interfere with study results. Male dogs used for breeding and pregnant or lactating female dogs were also excluded. Additionally, dogs were excluded if they had previously taken imepitoin or had family-related risk factors (e.g., toddlers in the home in the case of aggressive behavior towards people and aggression between dogs within home). Dogs were allowed to be given supplements or other products intended to reduce stress or anxiety (e.g., dog-appeasing pheromone, homeopathic remedies and pressure wraps) as long as they had been using them before starting the study and their use did not change throughout the study.

### 2.1. Study Design

**Figure 1:**
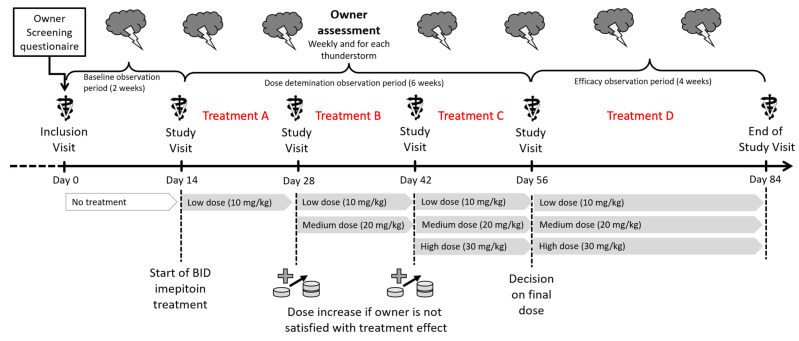
Schematic of study design. Physical examinations and laboratory work (serum biochemistry and complete blood count) were performed during the screening and end-of-study visits. At the end of each treatment period, the dosage was increased or maintained based on owners’ ratings of effect.

A preliminary screening was performed via an online questionnaire. Medical records of qualifying dogs were reviewed, and those meeting thew eligibility requirements were scheduled for an in-person assessment visit at the North Carolina State University College of Veterinary Medicine (NCSU-CVM). Following owner consent, a complete physical exam was performed, and baseline laboratory work (complete blood count, serum chemistry and urinalysis) was obtained. Dogs with normal laboratory work results, or with mild, stable systemic disease, were then enrolled in the study and began their baseline period. The study was divided into five periods: baseline (unmedicated) and treatment periods A to D (see Figure 1 for a schematic of the study design). Each period lasted a minimum of two weeks and required the owners to have witnessed and logged their dog’s response to a minimum of two storms. The owners were instructed to include any meteorological event related to storms (e.g., wind, rain, hail, thunder, etc.) as a storm if their dogs had historically reacted to them with signs of fear or anxiety. The treatment periods could be extended up to four weeks until two storms were logged. During treatment period A, all dogs were given imepitoin at approximately 10 mg/kg PO BID. After treatment period A, the dosage was either maintained or increased by approximately 10 mg/kg PO BID each period to a maximum dosage of approximately 30 mg/kg PO BID based on the treatment effect reported by owners. Treatment period D served as a stable dosing period and lasted four weeks, regardless of the number of storms logged during this time. All doses mentioned hereafter will be considered PO BID unless specified. Imepitoin is available as Pexion^TM^ in 100 and 400 mg tablets. The manufacturer provided dosing tables for 10, 20 and 30 mg/kg, where a weight range was assigned the equivalent number of tablets in half-tablet increments. For statistical analysis, dogs who received a range of 5 to 10.5 mg/kg were placed in the 10 mg/kg group, dogs who received a range of 10.51 to 25.5 mg/kg were placed in the 20 mg/kg group, and dogs who received a range of 25.51 and above in the 30 mg/kg group. No individualized behavioral or environmental plan was provided; owners were instructed to ensure their dog was kept in a safe and secure environment during storms, not punish them if they showed signs of fear, not to pay excessive attention to them during storms, and to ignore the storm noises. Systematic desensitization and counterconditioning to storms were not allowed during the study. At the end of the study, owners and dogs returned to the NCSU-CVM for an end-of-study physical exam and laboratory work (complete blood count, serum biochemistry and urinalysis).

### 2.2. Outcome Measures

#### 2.2.1. Efficacy

Treatment effect was evaluated via weekly owner-completed surveys (always online and completed by all owners regardless of the number of storms) and storm logs (SLs), which could be completed either online or on paper and returned to investigators at each appointment. In the weekly surveys, owners scored their dog’s anxiety in response to all storms over the preceding week using the Lincoln Sound Sensitivity Scale (LSSS). For the storm logs, the owners scored their dog’s storm anxiety using the Lincoln Canine Anxiety Scale (LCAS) every time they could observe their dog’s behavior during a storm that lasted a minimum of 15 min.

Both scales ask the owners to rate their dogs on 16 anxiety-related behaviors: running around, drooling, hiding, destructiveness, cowering, restlessness/pacing, aggressive behavior, freezing in place, vocalizing, panting, vomiting/defecating/urinating/ and or diarrhea, owner-seeking behavior, vigilance/scanning of the environment, bolting, shaking/ trembling and self-harm. On the LSSS (completed weekly), these behaviors are each given an intensity score (from 0—not present, to 5—extensive amount) and a frequency score (from 0—never, to 3—always); total instrument scores were calculated as the sum of the product of intensity and frequency for each behavior (with a possible range of 0–240). The LCAS (completed each time there was a storm) includes only the intensity ratings for each of the 16 behaviors, with a total possible range of 0–80. In both scales, lower scores indicate lower anxiety. In both surveys, owners also rated the treatment effect of imepitoin on their dog’s anxiety (worse effect to excellent effect). At the end of each treatment period, dosage adjustment decisions were made based on the owner-rated effect; dogs whose owners rated the treatment effect as excellent or good were maintained on their current dosage, and dogs with some effect or no effect had their dosage increased. The owners who indicated a worse effect were contacted for discussion regarding dosage and continuation in the study.

At the end of the study, owners completed a final online survey to determine their overall impression of the study drug’s effect on their dogs. The end-of-study survey consisted of eight statements regarding the use and effect of the study drug; owners rated their agreement with a 6-point scale (strongly agree to strongly disagree).

#### 2.2.2. Safety

Safety was evaluated by comparing the baseline laboratory work to laboratory work obtained at study completion or withdrawal. The owners were also asked to report any adverse events that occurred throughout the study. Specific questions regarding the presence or absence of adverse events were included in the weekly surveys. On the weekly survey, owners were asked the following: Over the past week, did your dog experience any adverse effects of the medication? (No, Yes, NA—Not started medication yet). If the owner selected Yes they received a list and were asked to select all that applied from the following: increased hunger, increased salivation, increased water intake, increased urination, increased activity, ataxia (wobbly, stumbling, difficulty walking or navigating stairs, or tripping when trying to walk), vomiting, diarrhea, irritability/aggressive behavior, decreased hunger, decreased activity or other (with a text box provided for a complete answer if selected). They were also asked if their dog had required any treatment for any adverse events. While owners were reminded at each visit to report any adverse events to us as they occurred, this served to ensure that no adverse events were missed.

### 2.3. Statistical Analysis

Demographic data were represented using descriptive statistics. Efficacy and safety data were analyzed using parametric and non-parametric tests as appropriate. To analyze weekly surveys and storm logs, data were simplified to the mean of the LSSS total score (weekly) or LCAS total score (storm logs) per treatment period for each dog, allowing for one response per treatment period per animal. Paired *t*-tests were run comparing the baseline value and the value at each treatment period. The effect of dosage on response was analyzed using Analysis of Covariance (ANCOVA), modeling LSSS and LCAS scores (separately) for each period as a function of the baseline value and dosage. To evaluate the effect of storm characteristics (i.e., the presence and absence of thunder/lightening) on efficacy, storm log data were also analyzed, including only storms that had thunder/lightening (excluding those with just wind or rain). To evaluate the effect of the number of storms dogs were exposed to on their weekly survey scores, a repeated-measures model (PROC MIXED) was fit to allow each dog to have a value for each week, while the dosage category and weekly number of storms varied. In order to evaluate the relationship between the owners’ categorical ratings of effect and weekly LSSS scores and LCAS scores, the data were analyzed using a repeated-measures model, allowing for effects of the week (nested in treatment period), treatment period, dose, and owner response to predict the log-transformed LSSS scores. Paired *t*-tests were used to evaluate the pre- and post-study lab work for continuous data. Where the data were below the lower limit of quantification for a test, distributions were analyzed using chi-square tests. The data were analyzed using Statistical Analysis System (SAS), Cary, NC, USA. Significance was set at *p* < 0.05, with correction for multiple comparisons noted where appropriate.

## 3. Results

### 3.1. Subjects

A total of 651 participants completed the preliminary screening questionnaire; 531 did not meet the inclusion criteria, and an additional 73 were excluded after review of their medical records. Examples of why dogs were excluded during the preliminary screening questionnaire include the following: already taking behavior-modifying drugs (n = 201), having family-related risk factors such as children under the age of eight living in the home (n = 76), having a history of aggression towards other pets in the home (n = 86) and/or towards people living in the home (n = 29), being anxious more than 50% of the time (n = 51) and/or having a history of epilepsy (n = 31). Thirty-two dogs were excluded for having an LSSS score of less than 30.

**Figure 2:**
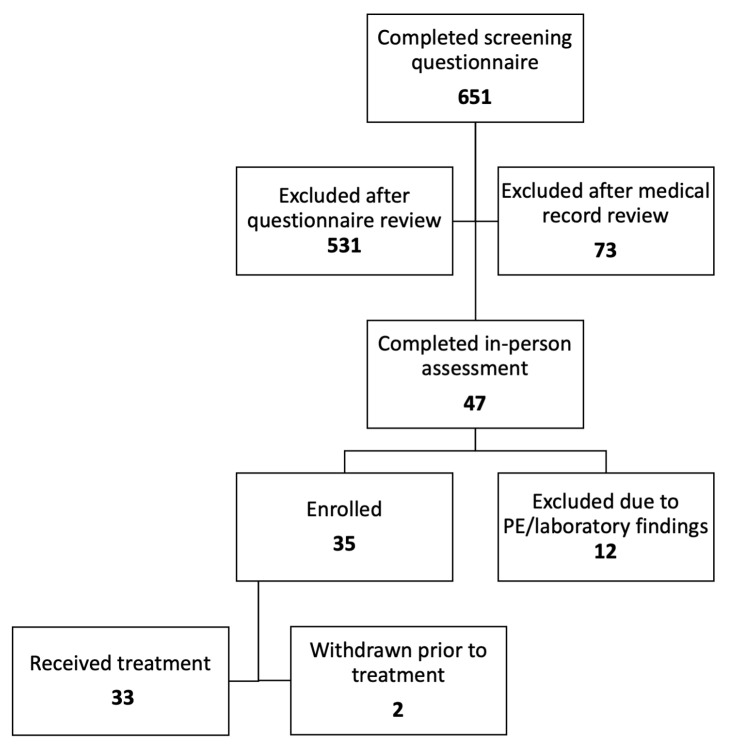
Enrollment flow diagram showing the number of dogs at each stage of enrollment.

Following the in-person assessment visit, 12 dogs were excluded due to physical exam or laboratory work abnormalities. A total of 35 dogs were initially enrolled in the study; two were withdrawn by the owners during the baseline period (one for an unrelated medical condition and one for not having sufficient number of storms). A total of 33 dogs received at least one dose of imepitoin during the study (see Figure 2 for flow diagram). The average age of participants was 6.8 ± 2.65 years (range 2–12 years) and the average weight was 20.5 ± 10.29 kg (range 6.5–38.1 kg). There were 19 females and 16 males enrolled, all were spayed or neutered and the female to male ratio was 1.06. No breed was over-represented.

**Figure 3:**
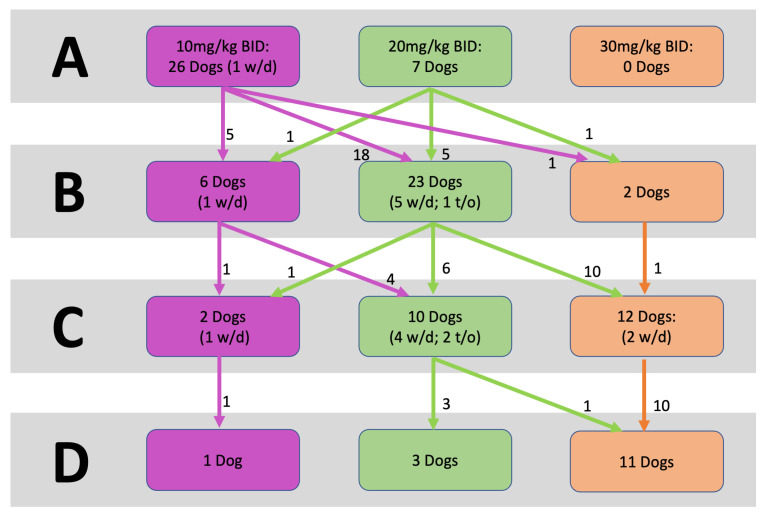
Flow diagram showing movement of enrolled dogs across treatment groups during each treatment period. (**A**) first dose determination period, (**B**) second dose determination period, (**C**) third dose determination period, (**D**) efficacy observation period. w/d = Withdrawal during that treatment period; t/o = timed out during that treatment period (reached the end of the study date without progressing to the next treatment period).

The mean duration of the study (including the baseline period) for participants was 11.5 ± 3.7 weeks (80.4 ± 25.7 days), ranging from 3.9 to 17 weeks (27 to 119 days). The movement of dogs across treatment groups during the study is shown in Figure 3. Dogs were exposed to an average of 0.9 ± 0.4 storms per week, ranging from 0.3 to 1.9 during the study. The mean duration of each treatment period and mean dosage over each period are shown in Table 1. Some dogs were given doses above 10.5 mg/kg BID in period A because of the initial dosing table design. In the subsequent periods, the dosing tables were adjusted to reflect the dosing categories described in Section 2.

### 3.2. Efficacy

#### 3.2.1. Weekly Surveys

The weekly survey efficacy data included the weekly score on the LSSS and the sum of the product of intensity and frequency scores for the 16 anxiety-related behaviors.

Paired *t*-tests between the baseline value and the value at each treatment period showed a significant lowering of the total score across all periods. The magnitude of the change increased each period; however, fewer dogs were represented in period C and D due to study withdrawals or reaching the end of study date prior to completion (Table 2).

**Figure 4:**
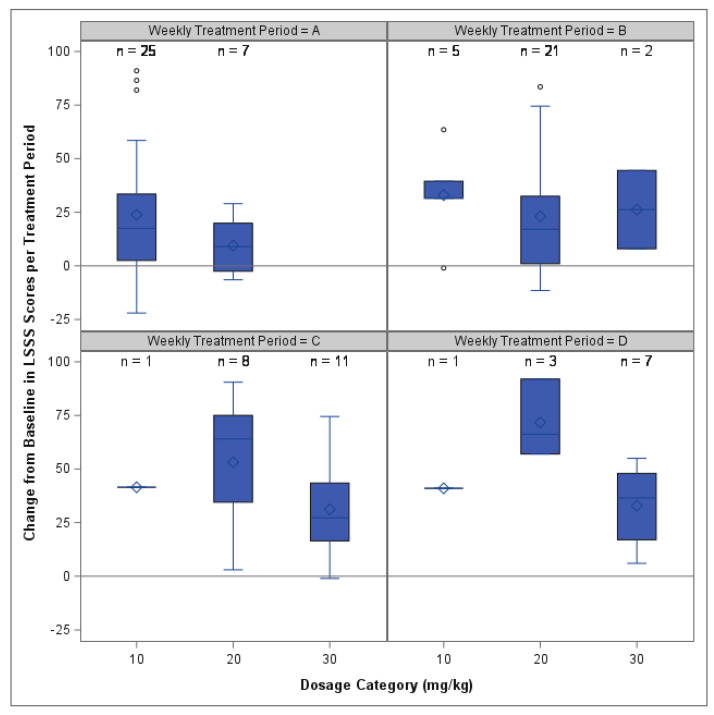
Box and whisker plots of the change from baseline in LSSS scores during each treatment period (**A**–**D**). Results within each treatment period are shown for each dosage group category (10, 20 or 30 mg/kg). Positive change from baseline indicates a lowering of anxiety scores, with higher numbers indicating greater improvement. The line at Y = 0 indicates no change in score. No significant pairwise differences were found between dosage groups in any period.

ANCOVA was used separately for each treatment period and showed an overall model dosage effect during periods A, C and D but no overall effects for period B. No pairwise differences were found between dosage groups for any treatment period (Supplementary Table S1). Changes from the baseline in the LSSS scores for each dosage group and period is shown in Figure 4.

#### 3.2.2. Storm Logs

The SL efficacy data included the LCAS score for each storm (the sum of the intensity scores for the 16 anxiety-related behaviors). Paired *t*-tests between the baseline value and the value at each treatment period showed a significant lowering of the total score across all periods (Table 3). Again, the magnitude of the change increased with each treatment period. The number of dogs with an LCAS score does not perfectly match those with a weekly LSSS score in certain cases where a storm log was not provided by the owner.

The number of dogs whose owners completed at least one storm log during that period is shown.

**Figure 5:**
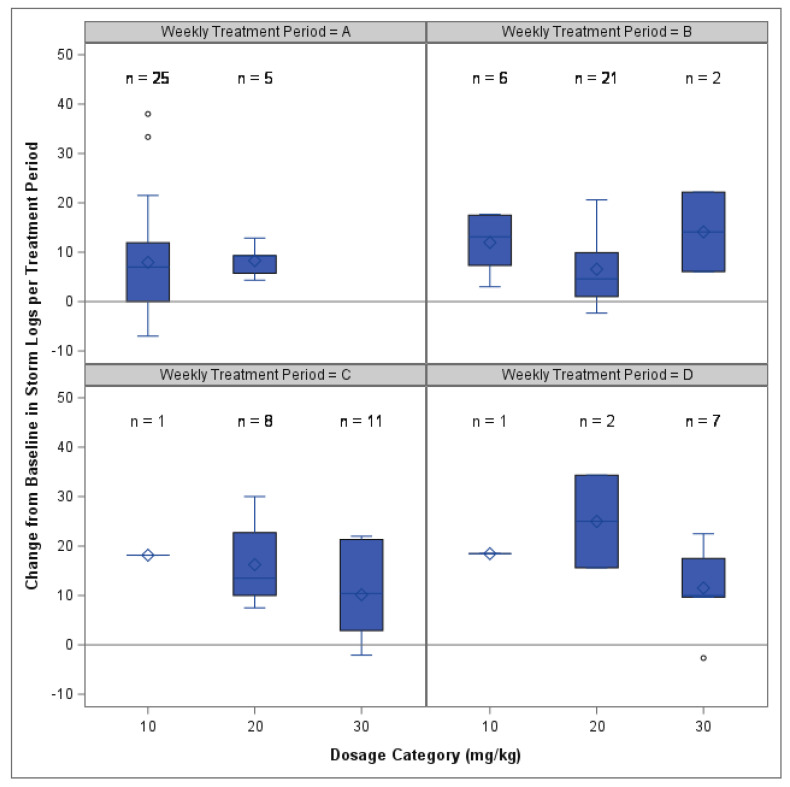
Box and whisker plots of the change in LCAS scores from baseline during each treatment period (**A**–**D**). Results within each treatment period are shown for each dosage group category (10, 20 or 30 mg/kg). Positive change from baseline indicates a lowering of anxiety scores. The line at Y = 0 indicates no change in the score. No significant pairwise differences were found between dosage groups in any period.

ANCOVA, run separately for each treatment period, found no detectable differences in the change in LCAS scores for the dosage groups during periods A or D, but detectable differences were found in periods B and C for the overall models. Despite the overall model effects, no pairwise differences were found between the dosage groups for any treatment period (Supplementary Table S2). Changes from the baseline LCAS score for each dosage group and period are shown in Figure 5.

When analyzing only those storms with thunder and/or lightening, paired *t*-tests between the baseline value and the value at each treatment period showed a significant lowering of the LCAS scores across all periods (Table 4), similar to that seen when analyzing all storms.

#### 3.2.3. Owners’ Categorical Rating of Effect Compared to LSSS and LCAS Scores

**Figure 6:**
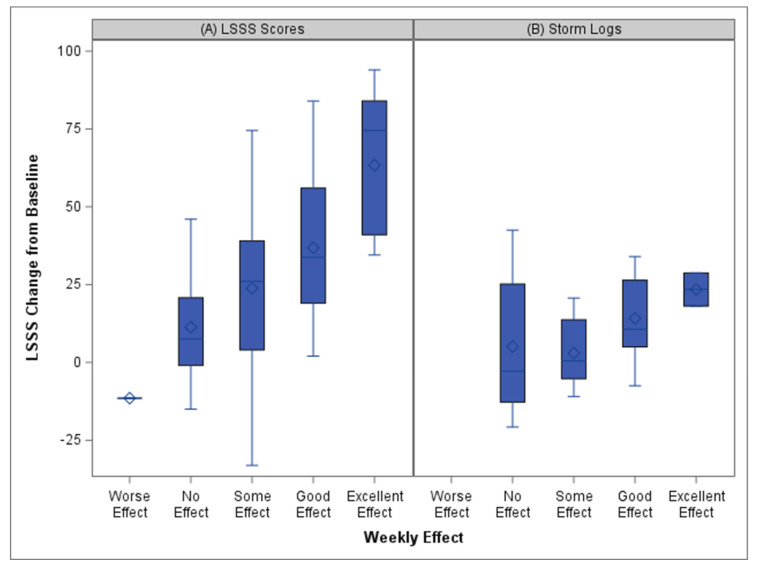
Box and whisker plots of the overall effect of medication. Owner ratings and the actual change in scores are shown for the (**A**) weekly LSSS from baseline scores and (**B**) storm log LCAS scores from baseline scores. Higher numbers indicate greater improvement.

An analysis of the relationship between the log-transformed weekly LSSS survey and the owners’ categorical ratings of the treatment effect was significant for the weekly dose category effect (F(2, 29) = 5.57, *p* = 0.009) and owners’ rating (F(4, 32) = 28.51, *p* < 0.0001). The nested effect allowed for multiple and varying numbers of weekly data points within each treatment period, but both the week-to-week changes and the treatment period changes were not statistically significant (treatment period: F(29, 43) = 1.17, *p* = 0.31; week-to-week: F(3, 53) = 1.61, *p* = 0.198). Pairwise comparisons are shown in Supplementary Table S3. Overall, there were significant differences in the LSSS scores for the categorical comparisons between excellent effect and all other categorical ratings. A similar analysis was performed for the relationship between the log-transformed storm log LCAS scores and the owners’ categorical ratings of treatment efficacy. This model was significant for only the owners’ categorical rating (F(4, 30) = 22.92, *p* < 0.0001). Pairwise comparisons are shown in Supplementary Table S4. Figure 6 shows the box and whisker plots of the changes from the baseline scores for each of the owners’ rating category for weekly surveys (A) and storm logs (B).

#### 3.2.4. Ease of Administration

On weekly surveys, owners rated the ease of administration of the medication to their dog. The majority of the owners (29/32, 91%) indicated it was easy to give their dog the medication during period A. This percentage decreased as the study progressed (27/30, 90% in B; 17/20, 85% in C; and 11/14, 78% in D). The main difficulty reported by owners in the comments was their dog’s ability to find the tablet hidden in food and spit it out, necessitating re-administration.

### 3.3. Safety

#### 3.3.1. Laboratory Work

Significant differences from baseline (screening) values were found for bicarbonate, cholesterol, chloride, creatinine, globulins, magnesium and sodium; when adjusted for multiple comparisons, only cholesterol, bicarbonate and globulins remained significantly different (See Supplementary Tables S4 and S5). There were no associated clinical signs, and the findings were not considered clinically significant.

#### 3.3.2. Adverse Events

No serious adverse events (AEs) were reported in any subject. Reported non-serious AEs were moderate or mild and were self-limiting or resolved shortly after medication discontinuation. Any adverse events (AEs) were reported in the majority of all treated dogs (27/33, 82%). AEs occurred more frequently in the 20 mg/kg group than the 10 or 30 mg/kg groups. Ataxia was the most common AE, followed by increased hunger and increased irritability/aggressive behavior. As patients could experience a given adverse event when taking different dosages of medication, the total case count at any dosage does not always equal the sum at each individual dosage. The distribution of AEs by dosage group and across all dosages is shown in Table 5.

#### 3.3.3. Withdrawals

Seventeen participants who started treatment withdrew before the end of the study. Four were unrelated to the study medication, and the rest cited AEs as the reason for withdrawal. Of those who withdrew due to AEs, the majority did so while taking 20 mg/kg (7/13, 54%), followed by 30 mg/kg (4/13, 31%) and 10 mg/kg (2/13, 15%). The AEs reported as reasons for withdrawal included ataxia (4), behavioral disinhibition (4), irritability/aggressive behavior (4), increased appetite (3), increased activity (3), increased anxiety (2), increased water intake (1) and gastrointestinal upset (1). Non-medication reasons for withdrawal included the taking of an exclusionary behavior-modifying drug (1), insufficient storms during an extended treatment period (1), and owners’ scheduling conflicts (2).

### 3.4. End-of-Study Survey

Results from the end-of-study survey are shown in Table 6. The end-of-study survey was completed by all 33 patients who took at least one dose of imepitoin.

## 4. Discussion

These results support our hypothesis that imepitoin at dosages of 10–30 mg/kg BID over a prolonged period during storm season would significantly decrease storm-associated anxiety and fear in dogs. Participants treated with imepitoin at these dosages had significant reductions in their LSSS and LCAS scores when compared to the baseline. No significant differences were found between dosage groups for any treatment period in either survey, indicating that an anxiolytic effect during storms can be seen using imepitoin at a range of 10 to 30 mg/kg and supporting an individual-dose titration approach.

When the data were analyzed to specifically look at thunderstorms (storms containing lightning and thunder), the results were similar, indicating that the anxiolytic effect of imepitoin was present with and without noise-related stimuli that owners could identify. Dogs might have heard thunder that was inaudible to their owners or have shown a response to non-auditory meteorologic stimuli such as changes in barometric pressure, static electricity, odors and flashes of lightning [1,9,10].

The safety profile of imepitoin in this study was consistent with previous studies of imepitoin at 30 mg/kg BID for up to three days for noise aversion [34]. No serious adverse events were reported, all AEs were moderate or mild and either self-limiting or resolved shortly after medication discontinuation, and no clinically relevant laboratory abnormalities were found. While there were no serious adverse events, at least one adverse event was reported in the majority of dogs. This percentage is higher than what has been reported in previous studies of imepitoin for behavioral disorders [8,34,35] and most studies for idiopathic epilepsy [38,39,40,41]. There is high variability in the report of AEs across these studies (ranging from 29% to 86%) in which dosages from 1 to 30 mg/kg BID were used. The interpretation of the number of AEs in the current study must consider the study duration, our pre-study discussions with owners about what signs to watch for and our proactive questioning about adverse events on the weekly surveys. This detailed investigation into the presence or absence of AEs may have captured milder AEs that were not reported in previous studies. The majority of the AEs were mild and self-limiting and were consistent with our previous study with imepitoin [8]. The most common adverse events were ataxia, increased hunger, irritability/aggression and behavioral disinhibition (captured as “other”). With regard to irritability/aggression (six cases), some of the dogs (3) had indications of previously existing reactivity toward other dogs either while on leash or in a kennel, but this was not screened for explicitly in our screening questionnaire. “New” irritability/aggressive behavior was reported in two dogs; this is equal to 6% and is consistent with reports seen in a retrospective study on diazepam in dogs [42]. Behavioral disinhibition also must be further qualified, as in many cases, this may have been a behavioral response to increased appetite. This category included dogs who took food off counters or showed other destructive behaviors. The incidence of AEs was highest in the 20 mg/kg group, followed by the 30 mg/kg and 10 mg/kg groups.

**Figure 7:**
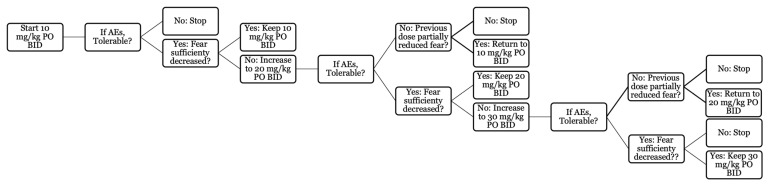
Imepitoin dose titration algorithm.

Seventeen dogs were withdrawn from the study, with 12 (36% of the dogs started on treatment) being withdrawn for a combination of adverse events and/or a perceived lack of effect. This may have been further affected by the number of storms and length of the study, as discussed below. It is the impression of the investigators that dogs who started on 10 mg/kg BID and experienced adverse effects and a lack of efficacy at that dose are unlikely to improve at higher dosages; however, dogs who responded at this dosage may show greater improvement at a higher dosage. Recommendations for the treatment algorithm are shown in Figure 7.

A limitation that affected our study and study results was the lack of consistent storms during the period (an average of less than one storm per week) and the subsequent extension of treatment periods. This may have contributed to the owners’ survey response burden. While not studied in depth in veterinary medicine, evidence from human clinical trial results show that increases in survey burden can affect study compliance [43,44]. For the owners who completed all four treatment periods, the average study duration was 14 weeks (±1.73 weeks; range 12–17). It may be particularly hard for the owners to continue their participation in the study and maintain compliance when storms are not consistently occurring; this is particularly true if any adverse events, even mild ones, are seen. Indeed, among our withdrawal cases, only one case withdrew despite owner reports of positive effects on anxiety. An additional limitation came from the original dosage tables, where some dogs were started on a dosage closer to 20 mg/kg BID than to 10 mg/kg BID. This affected one animal who had an adverse effect and whose dosage was lowered, while the others continued at the higher dosage. Efficacy at a lower dosage cannot be determined for these cases. Once this was noted and corrected, it did not occur again.

Another limitation was the reliance on the owners’ reported outcomes. The owners’ interpretation of the effect of the medication on their dog’s anxiety levels is subjective and can be influenced by a variety of factors, including, but not limited to, the study’s open-label design. As a robustness check, we asked for the owner report of the overall effect of the treatment in addition to the previously validated questionnaires and found a larger change in anxiety scores from the baseline for owners who reported a better overall effect, particularly for the excellent responses. We also found very similar results using the weekly scores (LSSS) and storm log scores (LCAS); thus, future studies could use only one of these outcome measures to decrease the owners’ study response burden.

## 5. Conclusions

In conclusion, this study’s results confirm what a previous placebo-controlled trial found: imepitoin may be a safe and effective medication for the reduction in fear- and anxiety-related behaviors during storms in dogs. We recommend starting imepitoin at a dosage of 10 mg/kg BID for the treatment of storm-related fear and anxiety in dogs during storm season and increasing as needed up to 30 mg/kg. As with other behavioral disorders in dogs, environmental management and behavior modification in addition to medication are recommended for the best results [13].

## Figures and Tables

**Table 1:** Mean duration (days) in each treatment period and mean dosage overall and for each dosing group within treatment periods. NA = Not applicable; no medication was administered during the baseline period.

Treatment Period	Number of Dogs at the Start of the Treatment Period	Mean Duration (Days ± Standard Deviation (SD))	Mean Dosage (mg/kg BID ± SD)	Mean Dosage in 10 mg/kg BID Group (mg/kg BID ± SD)	Mean Dosage in 20 mg/kg BID Group (mg/kg BID ± SD)	Mean Dosage in 30 mg/kg BID Group (mg/kg BID ± SD)
Baseline	33	17.48 ± 5.07	NA	NA	NA	NA
A	33	20.18 ± 8.02	12.24 ± 3.30	10.86 ± 2.03	17.35 ± 1.52	NA
B	31	17.52 ± 8.80	19.45 ± 5.28	10.16 ± 2.59	21.36 ± 2.42	25.40 ± 0.34
C	24	20.29 ± 12.52	24.58 ± 7.32	10.34 ± 4.66	19.97 ± 2.61	30.80 ± 2.47
D	15	24.90 ± 7.51	26.63 ± 7.45	7.04	19.81 ± 4.09	30.26 ± 2.75

**Table 2:** Paired *t*-test results for mean difference in LSSS score from baseline score during treatment periods. Number of dogs reflects those with completed weekly surveys in that treatment period (dogs who withdrew prior to a weekly survey are not included).

Period	Number of Dogs	Mean Difference (Base–Current)	Standard Error	Degrees of Freedom	t-Statistic	*p*-Value
A	32	21.03	4.83	31	4.35	0.0001
B	28	25.01	4.87	27	5.14	<0.0001
C	18	40.31	6.16	17	6.54	<0.0001
D	11	44.20	7.17	10	6.16	0.0001

**Table 3:** Paired *t*-test results comparing SL LCAS scores during each treatment period to baseline.

Period	Number of Dogs	Mean Difference (Base–Current)	Standard Error	Degrees of Freedom	t-Statistic	*p*-Value
A	30	8.01	1.84	29	4.35	0.0002
B	29	8.19	1.38	28	5.92	<0.0001
C	20	12.99	1.95	19	6.65	<0.0001
D	10	14.91	3.06	9	4.88	0.0009

**Table 4:** Paired *t*-test results comparing mean SL LCAS scores during each treatment period compared to baseline, restricted to storms containing lightning.

Period	Number of Dogs	Mean Difference (Base–Current)	Standard Error	Degrees of Freedom	t-Statistic	*p*-Value
A	29	7.80	1.86	28	4.2	0.0002
B	27	9.45	1.86	26	5.10	<0.0001
C	17	12.87	2.22	16	5.80	<0.0001
D	8	14.31	3.69	7	3.88	0.0060

**Table 5:** Distribution of adverse events across the study within each dosage group.

Adverse Event		10 mg/kg BID	20 mg/kg BID	30 mg/kg BID
Number of dogs administered this dose at any point in the study ^1^		N = 27 (%)	N = 32 (%)	N = 12 (%)
Increased hunger		0 (0)	12 (38)	1 (8)
Increased salivation		0 (0)	2 (6)	0 (0)
Increased water intake		0 (0)	3 (9)	1 (8)
Increased urination		0 (0)	1 (3)	0 (0)
Increased activity		0 (0)	4 (12)	0 (0)
Ataxia ^2^		6 (22)	9 (28)	2 (17)
Vomiting		1 (4)	2 (6)	0 (0)
Diarrhea		1 (4)	1 (3)	0 (0)
Irritability/Aggressive behavior		2 (7)	2 (6)	1 (8)
Decreased hunger		1 (4)	1 (3)	0 (0)
Decreased activity		1 (4)	2 (6)	0 (0)
Other ^3^ (all)		4 (15)	12 (38)	2 (17)
	Other: Behavioral disinhibition	1	8	1
	Other: Increased anxiety	2	3	1

Total case count at any dosage does not always equal the sum at each individual dosage because patients could experience a given adverse event when taking different dosages of medication. ^1^ Participants could experience more than 1 AE at a time. ^2^ Intensity of ataxia was characterized by owners as Mild (47%; 8/17 instances of ataxia), Moderate (41%; 7/17), Severe (6%; 1/17) or Not characterized (6%; 1/17). ^3^ In the other category, owners could list one or more behaviors; the category is separated based on behaviors noted in 4 or more cases; additional “other” behaviors included disorientation (2), panting (2), gagging (1), house soiling (1), regurgitation (1) and spinning (1).

**Table 6:** Results from the end of study survey.

	Agree	Disagree
The treatment was easy for me to administer to my dog	29/33, 88%	4/33, 12%
The treatment made my dog more relaxed and reduced my dog’s fear/anxiety during storms	19/32, 59%	13/32, 41%
The treatment made my dog more relaxed and reduced my dog’s fear/anxiety during other noises	15/30, 50%	15/30, 50%
After the first few doses, the treatment effect on my dog was predictable	23/32, 72%	9/32, 28%
Side effects (if any) were tolerable	16/28, 57%	12/28, 43%
Duration of the treatment effect was acceptable	22/27, 81%	5/27, 18%
The effect of the treatment for storms made me feel more relaxed	15/31, 48%	16/31, 52%
I would choose to continue giving this treatment to my dog	14/32, 44%	18/32, 56%

Owners of 33 dogs completed the questionnaires, but not all owners responded to all questions. Responses were collapsed into agree (strongly agree, agree and somewhat agree) and disagree (strongly disagree, disagree and somewhat disagree).

## Data Availability

The data that support the findings of this study are available from Boehringer Ingelheim Vetmedica GmbH, but restrictions apply to the availability of these data, which were used under license for the current study, and so are not publicly available. Data are, however, available upon reasonable request, and with permission of Boehringer Ingelheim Vetmedica GmbH, to Margaret Gruen.

## Supplementary Materials

The following supporting information can be downloaded at: https://www.mdpi.com/article/10.3390/ani14040545/s1, Table S1: Overall model effects and pairwise comparisons for weekly questionnaires for each period performed following ANCOVA; Table S2: Overall model effects and pairwise comparisons for storm log questionnaires for each period following ANCOVA; Table S3: Pairwise comparisons of the differences in LSSS score between categorical ratings of efficacy by owners; Table S4: Pairwise comparisons of the differences in LCAS score between categorical ratings of efficacy by owners; Table S5: Labwork values for selected serum biochemistry, complete blood count and urinalysis values; Table S6: Distribution analyses (Chi-square).
