# Supplementary material for: Pilot Study on the Efficacy and Safety of Long-Term Oral Imepitoin Treatment for Control of (Thunder)Storm-Associated Noise Phobia/Noise Aversion in Dogs Using an Individualized-Dose Titration Approach
Source: Animals (Basel). 2024 Feb 6;14(4):545. doi: 10.3390/ani14040545 (PMC10886229; doi:10.3390/ani14040545)
Supplement: Supplementary file 1 [file animals-14-00545-s001.zip › animals-2812361-supplementary.pdf]

Supplementary data tables

Supplementary Table S1. Overall model effects and pairwise comparisons for weekly questionnaires for each period performed following ANCOVA. There was no detectable difference between dosage groups in any period. The least squares means for each comparison are presented to show the patterns and numbers. Least squares means are adjusted to be the predicted mean value at the average of any covariate (here, baseline LSSS). The standard errors are quite large compared to the treatment differences, hence the lack of detectable differences.

Period	Overall F-test	P-value	Dosage Category	Number of Animals	LSSS Least Squares Mean	Std Error
A	F(2,30) = 4.83	0.0152	10	25	43.01	4.60
			20	7	47.84	8.816
B	F(3,24) = 2.78	0.0628	10	6	27.82	10.516
			20	22	38.88	5.130
			30	2	33.61	16.629
C	F(3, 14) = 3.48	0.0450	10	1	11.66	20.188
			20	8	13.37	7.505
			30	11	34.57	6.273
D	F(3, 7) = 4.61	0.0439	10	1	26.29	18.760
			20	3	-0.30	10.030
			30	10	38.11	7.409

Supplementary Table S2. Overall model effects and pairwise comparisons for storm log questionnaires for each period following ANCOVA. In period A, the dose categories did not differ statistically (F(2, 27)=1.75, p = 0.193. The table shows the least squares means and their standard errors, reinforcing that the groups did not differ. Least squares means are adjusted to be the predicted mean value at the average of any covariate. In period B, the dose categories were statistically significantly different (F(3, 25)= 10.63, p < 0.0001). In period B, the overall model was statistically significantly different (F(3, 25)= 10.63, p = 0.0001). However, none of the dose categories were statistically significantly different (F(2,25) = 1.83, p = 0.182). The coefficient for the baseline value is nearly one (0.915), indicating that as the baseline value increases, so does the Period B score. In period C, the overall model was statistically significant, but the dose differences are not (F(3, 16) = 3.32, p = 0.047). In period D, the sample size is smaller, the overall model is not statistically significant, and the dose differences are not different (F(3, 6) = 2.71, p = 0.138).

Period	Dosage category	Number of Animals	LCAS LSMean	Standard Error
A	10	25	16.59	1.76
	20	5	13.54	3.97

B	10	6	12.17	2.84
	20	21	17.35	1.60
	30	2	9.97	5.16
C	10	1	5.76	6.67
	20	8	7.94	2.36
	30	11	14.33	2.02
D	10	1	6.99	7.94
	20	2	1.45	5.13
	30	7	14.68	3.74

Supplementary Table S3. Pairwise comparisons of the differences in LSSS score between categorical ratings of efficacy by owners. Adjusted p-values are shown (using Tukey-Kramer adjustment for multiple comparisons).

Differences of Least Square Means							
Categorical Weekly Effect 1	Categorical Weekly Effect 2	Estimate	Standard Error	DF	t Value	Pr > |t|	Adj P
Excellent Effect	Good Effect	-1.793	0.27	32	-6.62	<.0001	<.0001
Excellent Effect	No Effect	-2.376	0.23	32	-10.08	<.0001	<.0001
Excellent Effect	Some Effect	-2.226	0.25	32	-8.96	<.0001	<.0001
Excellent Effect	Worse Effect	-2.883	0.96	32	-3.02	0.0050	0.037
Good Effect	No Effect	-0.582	0.25	32	-2.36	0.025	0.153
Good Effect	Some Effect	-0.433	0.24	32	-1.80	0.081	0.391
Good Effect	Worse Effect	-1.089	0.97	32	-1.13	0.267	0.790
No Effect	Some Effect	0.150	0.20	32	0.73	0.468	0.947
No Effect	Worse Effect	-0.507	0.95	32	-0.53	0.599	0.983
Some Effect	Worse Effect	-0.657	0.95	32	-0.69	0.493	0.956

Supplementary Table S4. Pairwise comparisons of the differences in LCAS score between categorical ratings of efficacy by owners. Adjusted p-values are shown (using Tukey-Kramer adjustment for multiple comparisons). SL=Storm log.

Categorical SL Effect 1	Categorical SL Effect 2	Estimate	Standard Error	DF	t Value	Pr > |t|	Adj P
Excellent effect	Good effect	-0.704	0.192	30	-3.66	0.0010	**0.0079**
Excellent effect	No effect	-1.429	0.169	30	-8.44	<.0001	**<.0001**
Excellent effect	Some effect	-1.196	0.173	30	-6.90	<.0001	**<.0001**
Excellent effect	Worse effect	-1.967	0.437	30	-4.50	<.0001	**0.0008**
Good effect	No effect	-0.725	0.144	30	-5.04	<.0001	**0.0002**
Good effect	Some effect	-0.492	0.134	30	-3.68	0.0009	**0.0076**
Good effect	Worse effect	-1.263	0.423	30	-2.98	0.0056	**0.0413**

No effect	Some effect	0.233	0.121	30	1.92	0.0646	0.3297
No effect	Worse effect	-0.538	0.419	30	-1.28	0.2092	0.7032
Some effect	Worse effect	-0.771	0.418	30	-1.85	0.0749	0.3677

Supplementary Table S5. Labwork values for selected serum biochemistry, complete blood count, and urinalysis values. Paired t-tests were performed to compare baseline and end of study values. P-values were adjusted for multiple comparisons within each panel. Analytes with significant differences in pre- vs. post-study values are presented in bold.

Analyte	N	Mean	StdErr	Minimum	Maximum	t-Value	DF	Prob-t	Adj p-value threshold
Albumin	32	-0.040	0.035	-0.5	0.3	-1.141	31	0.262	**0.0025**
Alkaline phosphatase	32	16.937	10.307	-18	324	1.643	31	0.110	
Alanine transaminase	32	10.344	6.920	-33	207	1.495	31	0.1451	
Amylase	32	49.125	27.279	-389	487	1.801	31	0.081	
Aspartate aminotransferase	32	3.812	2.141	-34	35	1.781	31	0.085	
Blood urea nitrogen	32	0.781	0.542	-4	10	1.441	31	0.160	
Calcium	32	0.181	0.076	-0.5	1.4	2.39	31	0.023	
**Cholesterol**	32	23.406	6.408	-27	133	3.652	31	**<0.001**	
Creatine kinase	32	28.25	37.178	-346	721	0.760	31	0.453	
Chlorine	32	-1.187	0.374	-6	3	-3.174	31	0.003	
Creatinine	32	0.094	0.040	-0.4	0.5	2.350	31	0.025	
Osmolality	32	-1.278	0.614	-7.9	5.9	-2.082	31	0.046	
**Globulin**	32	0.137	0.034	-0.1	0.7	4.030	31	**<0.001**	
Glucose	32	-1.500	1.582	-16	13	-0.948	31	0.351	
**Bicarbonate**	32	-1.656	0.455	-10	4	-3.641	31	**<0.001**	
Potassium	32	0.053	0.059	-0.9	0.8	0.902	31	0.374	
Lipase	32	20.343	13.643	-151	310	1.491	31	0.146	
Magnesium	32	0.062	0.029	-0.3	0.4	2.181	31	0.037	
Sodium	32	-0.844	0.336	-4	3	-2.509	31	0.018	
Phosphorous	32	1.11E-16	0.101	-1.2	1	1.1E-15	31	1	
WBC	32	-0.192	0.225	-2.64	2.49	-0.853	31	0.400	**0.017**
HCT	32	-0.937	0.595	-8.6	5.9	-1.576	31	0.125	
HGB	31	-0.081	0.183	-2.1	2.8	-0.442	30	0.662	
Urine specific gravity	25	0.002	0.0018	-0.017	0.029	1.244	24	0.225	**0.025**
Urine Ph	25	0.4	0.268	-2	3	1.494	24	0.148	

Supplementary Table S6. Distribution analyses (Chi-square).

	DF	χ^2	P-value
Bilirubin	2	0.137	0.934
GGT	4	0.137	0.998
